# Prevalence of major and minor electrocardiographic abnormalities and their relationship with cardiovascular risk factors in Angolans

**DOI:** 10.1016/j.ijcha.2022.100965

**Published:** 2022-02-09

**Authors:** Mauer A.A. Gonçalves, João Mário Pedro, Carina Silva, Pedro Magalhães, Miguel Brito

**Affiliations:** a Centro de Investigação em Saúde de Angola (CISA), Caxito, Bengo, Angola; b Faculdade de Medicina, Universidade Agostinho Neto, Luanda, Angola; c Centro de Estudos Avançados em Educação e Formação Médica (CEDUMED), Luanda, Angola; d Health and Technology Research Center (H&TRC), Escola Superior de Tecnologia da Saúde de Lisboa, Instituto Politécnico de Lisboa, Portugal; e CEAUL – Centro de Estatística e Aplicações, Faculdade de Ciências, Universidade de Lisboa, Portugal

**Keywords:** ECG, Major and minor electrocardiographic abnormalities, Cardiovascular risk factors, Angola

## Abstract

**Aims:**

To identify the prevalence of major and minor electrocardiographic abnormalities and their association with the main risk factors for cardiovascular disease in a population in the province of Bengo, northern Angola.

**Methods:**

A cross-sectional community-based study was conducted and a representative random sample stratified by sex and age was selected. In total, 2379 black individuals were included in the final analysis. A standard 12-lead ECG were recorded from all participants, analyzed and processed by the University of Glasgow software and coding by the Minnesota code.

**Results:**

22.3% of participants had minor electrocardiographic abnormalities and 4.58% major ECG abnormalities. The most common minor ECG abnormalities were abnormal T wave inversion, minor isolated ST abnormalities and premature beats. The most common major ECG abnormalities were Left ventricular hypertrophy with major ST-T abnormalities, Ventricular conduction defects and major Q-wave abnormalities. Hypertension, diabetes mellitus, hypercholesterolemia, alcohol consumption and smoking, were significantly associated with major and minor electrocardiographic abnormalities.

**Conclusions:**

In this study several participants had minor and major electrocardiographic abnormalities. Minor electrocardiographic abnormalities were more prevalent in men and major abnormalities in women. The electrocardiographic abnormalities had significant associations with the main cardiovascular risk factors.

## 1 Introduction

The electrocardiogram (ECG) is one of the most commonly used noninvasive tool in cardiology clinical practice, and it is the first complementary approach in the management of cardiac symptoms [1].

The importance of using the ECG in Medicine has been established for many years, and created a great advance and an innovative resource for the study of conditions related to heart disorders, with a great advantage in terms of diagnostic, accessibility and good accuracy [2]. The ECG has been used in cardiovascular epidemiology, due to its low cost, availability, safety of use and its ability to predict unfavorable outcomes in different populations [3], [4]. With recent methodological and technological advances, the use of ECG has become an important tool and widely used in population studies, mainly for identifying electrocardiographic abnormalities that predict major cardiovascular events [5].

Several surveys have described electrocardiographic characteristics in Africans living in Sub-Saharan Africa over the past 5 decades, and have reported the following distinctive ECG patterns: inverted and flattened T waves, ST elevation, axis deviation and high R wave amplitude [6], [7], [8], [9], [10], [11], [12], [13].

However, there is a scarcity of studies that associate electrocardiographic abnormalities to cardiovascular risk factors in African populations, especially in Sub-Saharan Africa, in contrast to several studies that have already established this relationship in Caucasian, Latin and African American populations [14], [15], [16], [17], [18], and there is no information in the medical literature on the electrocardiographic abnormalities and their relationship with cardiovascular risk factors in Angolans [10], [13], [19], [20].

Therefore, the aim of this study is to identify major and minor electrocardiographic abnormalities and their association with the main cardiovascular risk factors in a population of the province of Bengo, northern Angola, analyzed in the CardioBengo study [21].

## 2 Methods

### 2.1 Study design

The data presented herein was obtained from a community-based survey conducted in the catchment area of the Dande Health and Demographic Surveillance System (Dande-SVSD) located in the municipality of Dande, Bengo province, located 60 km north of Luanda, the capital of Angola. The CardioBengo was a cross-sectional, community-based survey conducted from September 2013 to March 2014. A representative random sample stratified by sex and age was selected, aged between 15 and 84 years old, the participants were assessed for sociodemographic, behavioral and physical characteristics. The study design has been described in detail elsewhere [21]. Participants with missing anthropometric values (n = 76), missing ECG (n = 4), and pregnant women (n = 116) were excluded from the analysis. Therefore, 2 379 black individuals were included in the final analysis.

## 3 Data collection

From all participants anthropometric data and information on age, education, alcohol and tobacco consumption was collected through a structured interview conducted by trained and certified interviewers. All the procedures followed the standardized protocol of the World Health Organization (WHO), based on the Surveillance Manual (STEPS) for Chronic Disease Risk Factors (central and expanded version 3.0) [22]. Measurements of blood pressure was performed with the automatic sphygmomanometer OMRON M6 Comfort (OMRON Healthcare Europe BV, Hoofddorp, The Netherlands), with the individual seated, and using an appropriate cuff size. Three readings were done at three-minute intervals. The mean value of the last two measurements was used to determine the blood pressure. Blood sugar was measured using a blood glucose meter ACCU-CHEK Aviva (Roche Diagnostic, Indianapolis, IN, USA) with ACCU-CHEK Aviva glucose reactive strips (Roche Diagnostic, Indianapolis, IN, USA). Total cholesterol in the blood was measured using a point-of-care device ACCUTREND Plus (Roche Diagnostic, Indianapolis, IN, USA) with ACCUTREND cholesterol reactive strips (Roche Diagnostic, Indianapolis, IN, USA).

Participants were considered diabetic if fasting blood glucose > 126 mg/dL or postprandial blood glucose > 200 mg/dL. Patients were classified as hypertensive if mean values of systolic blood pressure (SBP) ≥ 140 mmHg and / or diastolic blood pressure (DBP) ≥ 90 mmHg. Hypercholesterolemia was considered when a participant has total cholesterol levels > 240 mg/dL. Obesity was considered when body mass index ≥ 30 Kg/m^2^. Current smokers were defined as participants who reported smoking at least one cigarette per day.

## 4 Electrocardiographic measures

A standard 12-lead ECG and a rhythm strip were recorded at baseline from all participants using an AsCARD Mr. Grey V 201 12-channel electrocardiograph (ASPEL, Zabierzów, Poland). The examination was performed with the individual in resting supine position, respecting the participants rights to privacy. The exam was digitally recorded using the CARDIO TEKA v001 database software (ASPEL, Zabierzów, Poland). The digitally collected ECG tracings were sent electronically to the University of Glasgow Central Electrocardiography Laboratory, where they were analyzed and processed by the University of Glasgow software and coding by the Minnesota code (MC) [23]. The electrocardiographic global measurements were automatically calculated. The QT interval was corrected by the formula of Hodges, Bazett, Fridericia and Framingham, and the normal limits were previously published [24].

The Minnesota coding system was used to classify ECG tracings as having a major, minor or absence of abnormalities [23].

Abnormal ECGs were manually reviewed by two cardiologists to guarantee the quality of the coding.

### 4.1 Statistical analysis

Data was analysed considering gender stratification. Continuous variables were expressed as mean ± SD and categorical variables were expressed as number and proportions. Between-group differences were evaluated by the independent Student *t* test, test for difference between two independent population proportions or the χ2 analysis as appropriate. Bonferroni adjustments was used for multiple testing. Multivariate logistic regression analysis was performed to estimate the association between any minor or major electrocardiographic abnormality with age and sum of cardiovascular risk factors by sex. The adjusted odds ratios (ORs) were presented with 95% confidence interval (CIs). It was considered a significance level of 5%. Statistical analysis was conducted in IBM SPSS® (Statistical Package for the Social Sciences) version 26 and in R (version 4.0.5) software.

## 5 Results

Characteristics of the participants with valid electrocardiogram at baseline values of the 2 379 participants (880 males and 1499 females) and stratified by gender is showed in Supplementary Table 1A. Average (±SD) of the age was 35.0 ± 14.5 years old and 63% of the sample were female.

In Table 1 it is presented the prevalence of electrocardiographic abnormalities according to gender. The minor ECG abnormalities at baseline were found on 22.3% of the individuals (30.9% male, 17.3% female) and major ECG abnormalities were found on 4.58% of the individuals (3.2% male, 5.4% female). The most common minor ECG abnormalities were abnormal T wave inversion (n = 167; 7.03%). The second more frequent minor abnormality was minor isolated ST abnormalities (n = 101; 4.2%), followed by premature beats (n = 78, 3.27%). The most common major ECG abnormalities were LVH with major ST-T abnormalities (n = 55; 2.31%). It was followed by ventricular conduction defect (n = 21; 0.88%) and major Q-wave abnormalities (old myocardial infarction) (n = 17; 0.71%). The prevalence of electrocardiographic abnormalities according to gender is showed in detail in Supplementary Table 1B.

**Table 1:** Prevalence of minor and major electrocardiographic abnormalities stratified by gender.

	**No. (%) of Participants**			
**Minnesota Code abnormalities**	**Male** **(n = 880)**	**Females** **(n = 1499)**	***p***	**All**
**Minor abnormalities**	**272 (30.9)**	**259 (17.3)**	<0.001	**531 (22.3)**
Sinus bradycardia	22 (2.5)	2 (0.13)	<0.001	24 (1.01)
First degree AV block	10 (1.14)	7 (0.47)	0.061	17 (0.72)
Incomplete IV blocks	8 (0.91)	8 (0.53)	0.280	16 (0.67)
Ectopic atrial rhythm	10 (1.14)	13 (0.87)	0.515	23 (0.97)
High T-wave amplitude	7 (0.8)	22 (1.47)	0.149	29 (1.22)
Low QRS voltage in limbs leads	3 (0.34)	14 (0.93)	0.096	17 (0.72)
Right atrial enlargement	3 (0.34)	4 (0.27)	0.748	7 (0.29)
Poor R progression	1 (0.11)	0 (0.0)	0.190	1 (0.04)
Minor isolated Q,QS waves	2 (0.23)	4 (0.27)	0.849	6 (0.25)
Minor isolated ST abnormalities	44 (5.0)	57 (3.8)	0.161	101 (4.25)
Abnormal T wave inversion	28 (3.18)	139(9.27)	<0.001	167(7.01)
Wandering pacemaker	1 (0.11)	0 (0.0)	0.190	1 (0.04)
Left axis deviation	11 (1.25)	13 (0.87)	0.368	24 (1.01)
Minor QT abnormalities	4 (0.45)	7 (0.47)	0.968	11 (0.46)
Premature beats	26 (2.95)	52 (3.46)	0.497	78 (3.27)

**Major abnormalities**	**N = 28 (3.2)**	**N = 81 (5.4)**	0.012	**109 (4.6)**
AV conduction defect	1 (0.11)	1 (0.07)	0.704	2 (0.08)
Ventricular conduction defect	5 (0.56)	16 (1.06)	0.208	21 (0.88)
Left ventricular hypertrophy with major ST-T abnormalities	12 (1.36)	43 (2.87)	0.018	55 (2.31)
Right Ventricular Hypertrophy	1 (0.11)	3 (0.2)	0.617	4 (0.17)
Major Q-wave abnormalities (old MI)	7 (0.8)	10 (0.67)	0.718	17 (0.72)
Major isolated ST-T abnormalities	6 (0.68)	2 (0.13)	0.025	8 (0.34)
Major QT prolongation index (QT index ≥ 116%)	2 (0.23)	0 (0.0)	0.064	2 (0.08)
Arrhythmias	2(0.23)	2(0.13)	0.590	4(0.16)

*p*-values were obtained by the test for the difference between two independent population proportions.

The relationship between cardiovascular risk factors and ECG abnormalities is presented in Table 2. There was a significant association between minor and major abnormalities and individuals with hypertension, diabetes, hypercholesterolemia, obesity, alcohol and tobacco users.

**Table 2:** Association analysis between cardiovascular (CDV) risk factors and ECG abnormalities.

**Characteristics**	Normal ECG n = 1739	Minor ECG abnormalities n = 531	Major ECG abnormalities n = 109	All n = 2 379	p*
**Hypertension**					
Yes	289 (16.6)	94 (17,8)	72 (66,1)	455 (19.1)	
No	1450 (83.4)	434 (82,2)	37 (33.9)	1921 (80.9)	<0.001

**Diabetes mellitus**					
Yes	164 (9.5)	49 (9.3)	19 (17.4)	232 (9.8)	
No	1571 (99.5)	480 (90.7)	90 (82.6)	2141 (90.2)	0.022

**Obesity**					
Yes	452 (26.0)	149 (28.1)	34 (31.2)	635 (26.7)	
No	1288 (74.0)	381 (71.1)	75 (68.8)	1744 (73.3)	<0.001

**Hypercholesterolemia**					
Yes	268 (20.0)	92 (22.7)	30 (32.3)	390 (21.2)	
No	1075 (80.0)	314 (77.3)	63 (67.7)	1452 (78.8)	0.014

**Smoking**					
Yes	97 (5.6)	31 (5.9)	18 (16.7)	146 (6.2)	
No	1639 (94.4)	491 (94.1)	90 (83.3)	2220 (93.8)	<0.001

**Alcohol Consumption**					
Yes	635 (36.6)	165 (31.3)	49 (45.0)	849 (35.8)	
No	1100 (63.4)	362 (68.7)	60 (55.0)	1522 (64.2)	0.011

**Physical activity^#^**					
Yes	1649 (99.4)	495 (99.4)	89 (98.9)	2233 (99.4)	
No	19 (0.6)	3 (0.6)	1 (1.1)	20 (0.6)	0.835

# physically active vs sedentary: *p-values obtained using Qui-Square test

**Fig. 1:**
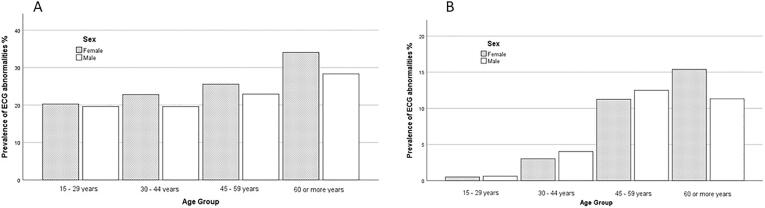
(A) Prevalence of minor electrocardiographic abnormalities distribution by gender and age group; (B) prevalence of major electrocardiographic abnormalities distribution by gender and age group.

In Fig. 1 it was showed the prevalence of minor (A) and major (B) electrocardiographic abnormalities distribution stratified by gender and age group. It is observed that the prevalence of minor abnormalities increases with advancing age in both sexes (female 4% and male 2%) and all minor electrocardiographic abnormalities were more prevalent in the elderly. Regarding major abnormalities, there was found an increase as age advances in women (5%) and an increase up to 59 years in men (6%), noting a slight decrease in men over 60 years (1.5%).

**Fig. 2:**
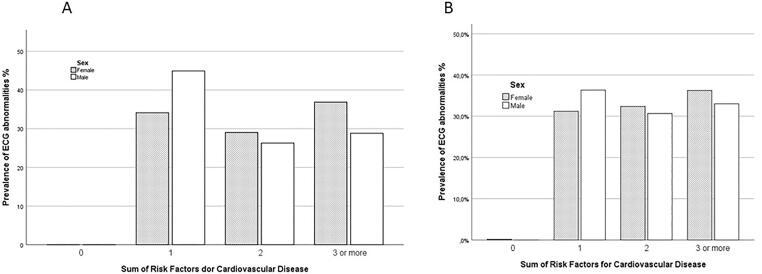
(A) Prevalence of minor ECG abnormalities distribution by Sum of Risk Factors for CVD and gender (B) Prevalence of major ECG abnormalities distribution by Sum of Risk Factors for CVD and gender.

In Fig. 2 it is showed the relation between sum of cardiovascular risk factors and the prevalence of minor (A) and major (B) electrocardiographic abnormalities stratified by gender. It is observed that in the percentage of minor and major ECG abnormalities was higher in individuals who presented only 1 risk factor (45% and 36%), while in women this percentage was higher in the group with 3 or more risk factors (37% and 36%).

**Supplementary figure 1:**
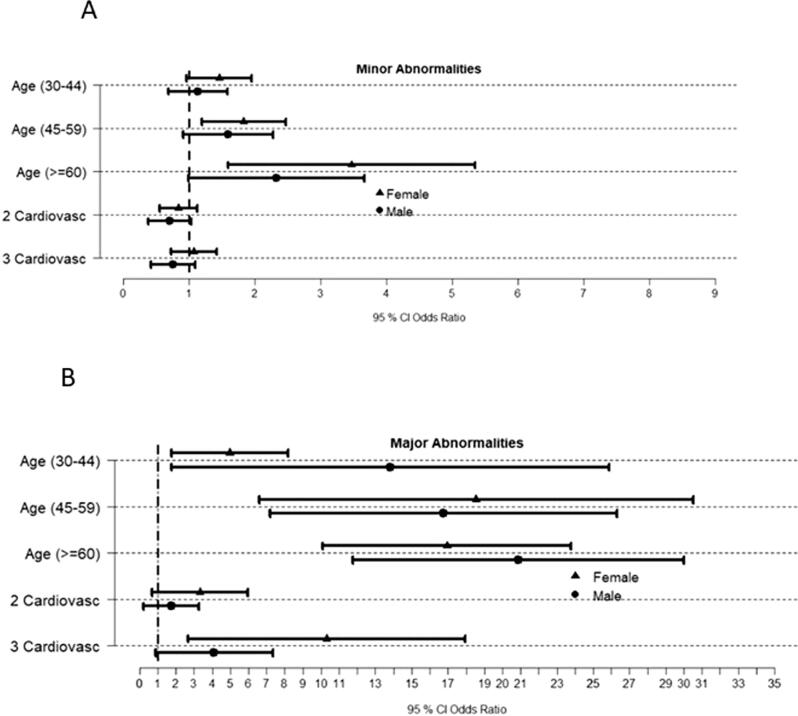
Odds ratio for the presence of any minor electrocardiographic abnormality (A) or major electrocardiographic abnormality (B) in men and women stratified by age and cardiovascular risk factors. Points represents the odds ratio and bars represent their 95% confidence interval (the reference parameters for age group is “15 to 29” years and for cardiovascular risk factors is “1 cardiovascular risk factor”)

The Supplementary Fig. 1 gives information about odds ratio (OR) for the presence of any minor (A) and major (B) electrocardiographic abnormality in men and women stratified by age and cardiovascular risk factors. It is observed that the odds of having any minor and major ECG abnormalities increased with all age groups (30–44, 45–59 and 60 or greater) in both men and women compared to the reference group “15–29” years. The OR for women with three or more risk factors for having minor ECG abnormalities was 1.1 (95% CI 0.7 to 1.4) compared to the reference group “1 cardiovascular risk factor” and it was 0.7 for men (95% CI 0.4 to 1.1). The reveals the OR for women with three or more risk factors for having major ECG abnormalities was 10.5 (95% CI 2.6 to 18) compared to the reference group “1 cardiovascular risk factor” and it was 4.1 for men (95% CI 0.9 to 7.4). The OR for women with Hypertension for having major ECG abnormalities was 5.5 (95% CI 4.6 to 10.5) and it was 5.3 for men (95% CI 1.1 to 10.3).

The OR for women with three or more risk factors for having minor ECG abnormalities was 1.1 (95% CI 0.7 to 1.4) compared to the reference group “1 cardiovascular risk factor” and it was 0.7 for men (95% CI 0.4 to 1.1). The OR for women with three or more risk factors for having major ECG abnormalities was 10.5 (95% CI 2.6 to 18) compared to the reference group “1 cardiovascular risk factor” and it was 4.1 for men (95% CI 0.9 to 7.4). The OR for women with Hypertension for having major ECG abnormalities was 5.5 (95% CI 4.6 to 10.5) and it was 5.3 for men (95% CI 1.1 to 10.3) (Supplementary Fig. 1).

## 6 Discussion

The present study showed for the first time that major electrocardiographic abnormalities have a prevalence of 4.58% and minor electrocardiographic abnormalities a prevalence of 22.3% in Angolans from municipality of Dande, Bengo province.

In this study, minor abnormalities were more prevalent in men (30.9% vs 17.3%) which is in line with most existing studies [14], [25]. Women had a higher prevalence of major abnormalities (3.2% vs 5.4%), which differs from most existing studies [14], [15], [16], [17], [25].

The most common minor ECG abnormalities were abnormal T wave inversion and were more frequent in women (3.81% vs 9.27%, *p* < 0.001). The second most frequent minor alterations were isolated ST abnormalities and were more frequent in men (5.0% vs 3.8%, *p* = 0.161).

These alterations are in line of findings published in a Systematic Review about association of traditional cardiovascular risk factors with development of major and minor electrocardiographic abnormalities, which showed that the most frequent minor abnormalities in blacks were isolated minor nonspecific ST-segment and T-wave abnormalities (NSSTTA) [26]. In same line, a study carried out in Cameroon showed that T-wave abnormalities were the more frequent minor abnormality found with 20.9% [27].

A Systematic Review about Clinical Significance of Minor Nonspecific ST-Segment and T-Wave Abnormalities in Asymptomatic Subjects, revealed that for any or isolated minor NSSTTA, the prevalence seems to be greater in women than men, what is in line with our results [28]. Data from others studies suggest that minor NSSTTA are more prevalent in women, and they also may be associated with greater increased risk for future CVD and coronary heart disease (CHD) events than in men [29], [30]. Ashley et al. (2000) admit that the reason for the increased prevalence of NSSTTA in middle-aged and older women may be the effect of losing the protective effect of estrogen, which acts as a vasodilator and antioxidant, and appears to influence cardiac natriuretic peptides by through the renin-angiotensin system [31], [32].

Several studies have examined the risk associated with isolated NSSTTA and the subsequent incidence of mortality from CHD and CVD in asymptomatic white people and found that smaller isolated NSSTTAs are significantly associated with mortality from CHD and CVD, regardless of traditional risk factors, but there are scarce data about the risk for incident CVD associated with minor NSSTTA in blacks compared with whites [28], [33].

The third most frequent minor alteration was premature beats (atrial premature beats, 2.02% and ventricular premature beats, 1.26%) and were more frequent in women, but with no statistically significant difference (2.95% vs 3.27%, *p* = 0.497). These data are in line with a Dutch study that revealed similar data [34] e differ from the results of other studies [10], [13], [17].

In general, the high prevalence of premature beats is directly related to increasing age due to the increase in cardiovascular diseases in this population [35].

In what concerns major ECG abnormalities, the most frequent, both in men and women, were left ventricular hypertrophy with major ST-T abnormalities and were more frequent in women (1.36% vs 2.86%, *p* = 0.018). A Cameroonian study showed that, LVH was the more frequent major abnormality found with 16.2% [27]. Study performed in Brazil, revealed that major abnormalities were more prevalent in black men, mainly due to isolated ST-T abnormalities and LVH with major ST-T abnormalities [15]. Healy et al. (2016), reveals that LVH was the triple in black men, which does not agree with the results of this study, in which LVH was twice as high in women [26].

Higher QRS voltage is more frequently observed in healthy black adults and the ignorance of these patterns can lead to incorrect diagnoses or therapeutic neglect [36], [37].

The second more frequent major ECG abnormalities was ventricular conduction defect (complete or intermittent intraventricular blocks) and were more frequent in women, but with no statistically significant difference (0.56% vs 1.06%; *p* = 0.208). A study carried out in Latinos and Polish found similar findings [17], [38], while studies in South Africans, middle-aged Africans, French and Americans found different results [10], [13], [18], [39]. The ventricular conduction defect in this sample can be explained by the higher proportion of individuals with hypertension as this condition is known to be harmful for the ventricular conduction system [3], [4], [40], [41].

The third most common abnormality of our study was major Q-wave abnormalities (old MI present), and our study showed that the major Q-wave abnormalities were higher in men, but with no statistically significant difference (0.8% vs 0.67%, *p* = 0.718). A study conducted in urban South Africans showed that the “ischemic” changes were more prominent in men than in women [13]. Other cohorts showed different results. Zerkiebel et al. (2000) reported in a Middle-aged African Population in the Seychelles Islands a prevalence of “old MI present“ of 3.8% in men [95% CI 2% to 6.4%] and 0% in women [one-sided 95% CI 0% to 0.8%] [10]. Pinto-Filho et al. (2017) revealed in a study in Brazil a prevalence of major Q waves of 3.3% in men and 1.3% in women [15].

In the present study, the hypertensive individuals had 17.8 and 66.1% of minor and major ECG abnormalities, respectively. Several studies have shown that hypertension is the risk factor most associated with electrocardiographic abnormalities, mainly the changes in the NSSTTA [3], [4], [29], [40], [41] and LVH with major ST-T in Africans [9], which is in agreement with the main changes found in this study.

Of the 9.8% of diabetics identified in the study, 9.3% had minor abnormalities and 17.4% had major abnormalities. Study carried out in Brazil have shown 6.6% of minor ECG abnormalities and 25% of major ECG abnormalities in diabetics [16]. The prevalence of ECG abnormalities in this study, specially T-wave abnormalities, are comparable to the estimates reported from Africans diabetics living in Africa [27], [42], [43] and Chinese adults [41].

It was found that in this study 8.1% of obese individuals had electrocardiographic abnormalities. Liao et al. revealed that a high BMI was associated with a high prevalence of minor and major ECG abnormalities [40].

In this research, there was an association between hypercholesterolemia and electrocardiographic abnormalities, an association established in several studies in several countries [3], [4], [16], [40], [44].

Smokers had 5.9% minor ECG abnormalities and 16.7% had major ECG abnormalities and showed a positive association with the presence of electrocardiographic abnormalities. Results of other research have also found an association between smoking and electrocardiographic abnormalities [4], [40], [45]. Study conducted in the US revealed that smokers had more major ECG abnormalities, especially major Q waves, than has been observed in the general population [46]. Another research has revealed that individuals who consume more than 25 cigarettes a day have an increased risk of acute myocardial infarction compared to those who never smoke [47].

In this study, the alcohol consumers had 31.3% of minor abnormalities and 45.0% had major abnormalities. There was an association between alcohol consumption and electrocardiographic changes, which is in line with a study that reported this association [16]. While several studies have not shown this association [3], [4], [48]. These conflicting results can be explained by the difficulty in assessing alcohol consumption between different surveys. In this study, the WHO Manual STEPS was used [22].

Regarding physical activity, there was no association between physically active individuals and electrocardiographic abnormalities. Similar result to other studies, which did not find this association [16].

About cardiovascular risk factors and their association with the major electrocardiographic abnormalities, several studies have shown that the prevalence of abnormalities on the ECG was very higher in those with traditional cardiovascular risk factors [14], [16], [17], [26].

Auer et al. (2012), in their study on Association of major and minor ECG abnormalities with coronary heart disease events, revealed that the presence of major or minor ECG abnormalities at baseline was associated with CHD risk during follow‑up, independent of traditional cardiovascular risk factors, and the findings of the study suggest that the presence of ECG abnormalities, should be given consideration as they may indicate an adverse underlying cardiovascular risk profile and the prevalence of major and minor ECG abnormalities increases substantially with age [3].

Previous studies have reported a higher prevalence of classical risk factors in subjects with minor NSSTTA [29]. However, it is unclear whether the development of prognostically significant ECG abnormalities is solely attributable to aging or heritable factors or whether modifiable traditional risk factors are associated with their development prospectively. If the latter is true, then it is possible that earlier intervention on modifiable risk factors could prevent the development of major and minor ECG abnormalities, and thus the risk that they represent [28].

In this study, the OR of having minor ECG abnormalities, significantly increased with age in both men and women, and presence of three or more cardiovascular risk factors was significant only in women. Regarding the odds ratio of having major ECG abnormalities, in both men and women, significantly increased with age and the presence of 3 or more cardiovascular risk factors, which is in line with several studies that found similar results [14], [15].

There are a number of implications arising from our findings. First, some of the observed ST-segment and T-wave abnormalities could be variations presumably benign, but also may resemble those of malignant disease, as previously described in African origin individuals [36], [37], [49].

The main major abnormalities found, LVH with major ST-T abnormalities, although it may be a presumably benign pattern in blacks, is considered an established marker for coronary heart disease and was associated with increased risk of cardiovascular-related mortality [50], as well as the prevalence of the Ventricular conduction abnormalities and main Q wave, which can translate into a prevalent myocardial infarction, are findings of this study that should make us to reflect and deepen our knowledge and research on its impact on the risk of cardiovascular morbidity and mortality in the Angolan population.

It is important to highlight a limitation of this dataset, the cross-sectional study design, as the causal relationship cannot be established. Also, the low representativeness of individuals over 64 years of age. Another limitation is the fact that the ECGs were obtained only once at the beginning of the study, ECG criteria can be dynamic and could be more significant if several ECGs were obtained at different time points. However, this study has other strengths: i) it is one of the first population-based study conducted in Angola, with a representative sample randomly selected; ii) is the largest study to date, to identify ECG abnormalities in an indigenous population of sub-Saharan Africa; iii) ECG collection, recording and measurement were standardized, and the exams were performed by a single trained technician.

## 7 Conclusions

This is a pioneering study in Angola and the largest study to date on ECG abnormalities in black individuals residing in Africa.

In this study, several had minor electrocardiographic abnormalities, and contrary to other studies, minor abnormalities were more prevalent in men and major abnormalities in women.

The most common minor ECG abnormalities were abnormal T wave inversion and minor isolated ST abnormalities. The most common major ECG abnormalities were LVH with major ST-T abnormalities and major Q-wave abnormalities.

There was a significant association between minor and major abnormalities and individuals with hypertension, diabetes, hypercholesterolemia, obesity, alcohol and smokers.

Most of these electrocardiographic abnormalities, namely forms of T-wave and/or ST changes and LVH with major ST-T abnormalities, which are more prevalent in healthy black adults populations, can be presumably benign, and their knowledge can avoid certain diagnostic interventions or in making unnecessary therapeutic decisions.

## CRediT authorship contribution statement

**Mauer A.A. Gonçalves:** Conceptualization, Formal analysis, Investigation, Methodology, Project administration, Writing – original draft. **João Mário Pedro:** Conceptualization, Data curation, Formal analysis, Funding acquisition, Investigation, Writing – review & editing. **Carina Silva:** Formal analysis, Investigation, Methodology, Supervision, Validation, Writing – review & editing. **Pedro Magalhães:** Conceptualization, Formal analysis Investigation, Methodology, Project administration, Supervision, Validation, Writing – review & editing. **Miguel Brito:** Conceptualization, Data curation, Formal analysis, Funding acquisition, Investigation, Methodology, Project administration, Supervision, validation, Writing – review & editing.

## Declaration of Competing Interest

The authors declare that they have no known competing financial interests or personal relationships that could have appeared to influence the work reported in this paper.

## References

[1] Blackburn H., Keys A., Simonson E., Rautaharju P., Punsar S. (1960). The Electrocardiogram in Population Studies. Circulation.

[2] P.W. Macfarlane, A. van Oosterom, O. Pahlm, P. Kligfield, M. Janse, J. Camm (Eds.), Comprehensive Electrocardiology, 2.a ed., 2010.

[3] Auer R., Bauer D.C., Marques-Vidal P., Butler J., Min L.J., Cornuz J. (2012). Association of major and minor ECG abnormalities with coronary heart disease events. JAMA.

[4] Denes P., Larson J.C., Lloyd-Jones D.M., Prineas R.J., Greenland P. (2007). Major and minor ECG abnormalities in asymptomatic women and risk of cardiovascular events and mortality. JAMA.

[5] D’Agostino R.B., Vasan R.S., Pencina M.J., Wolf P.A., Cobain M., Massaro J.M., Kannel W.B. (2008). General cardiovascular risk profile for use in primary care: the Framingham Heart Study. Circulation.

[6] Somers K., Rankin A.M. (1962). The Electrocardiogram in healthy East African (Bantu And Nilotic) men. Heart.

[7] Walker A.R.P., Walker B.F. (1969). The bearing of race, sex, age, and nutritional state on the precordial electrocardiograms of young South African Bantu and Caucasian subjects. Am. Heart J..

[8] Ashcroft M.T., Miller G.J., Beadnell H.M.S.G., Swan A.V. (1971). A comparison of T-wave inversion, S-T elevation, and RS amplitudes in precordial leads of Africans and Indians in Guyana. Am. Hear. J..

[9] Araoye M.A. (1994). The physiological basis of ST-T variations in the electrocardiogram: a review. Afr. J. Med. Med. Sci..

[10] Zerkiebel N., Perret F., Bovet P., Abel M., Jaggy C., Paccaud F., Kappenberger L. (2000). Electrocardiographic findings in a middle-aged African population in the Seychelles islands. J. Electrocardiol..

[11] Jaggy C., Perret F., Bovet P., van Melle G., Zerkiebel N., Madeleine G. (2000). Performance of classic electrocardiographic criteria for left ventricular hypertrophy in an African population. Hypertens (Dallas, Tex 1979).

[12] Sutherland S.E., Gazes P.C., Keil J.E., Gilbert G.E., Knapp R.G. (1993). Electrocardiographic abnormalities and 30-year mortality among white and black men of the Charleston Heart Study. Circulation.

[13] Sliwa K., Lee G.A., Carrington M.J., Obel P., Okreglicki A., Stewart S. (2013). Redefining the ECG in urban South Africans: Electrocardiographic findings in heart disease-free Africans. Int. J. Cardiol..

[14] Denes P., Garside D.B., Lloyd-Jones D., Gouskova N., Soliman E.Z., Ostfeld R., Zhang Z.-M., Camacho A., Prineas R., Raij L., Daviglus M.L. (2013). Major and Minor Electrocardiographic Abnormalities and Their Association With Underlying Cardiovascular Disease and Risk Factors in Hispanics/Latinos (from the Hispanic Community Health Study/Study of Latinos). Am. J. Cardiol..

[15] Pinto-Filho M.M., Brant L.C.C., Foppa M., Garcia-Silva K.B., Mendes de Oliveira R.A., de Jesus Mendes da Fonseca M., Alvim S., Lotufo P.A., Mill J.G., Barreto S.M., Macfarlane P.W., Ribeiro A.L.P. (2017). Major Electrocardiographic Abnormalities According to the Minnesota Coding System Among Brazilian Adults (from the ELSA-Brasil Cohort Study). Am. J. Cardiol..

[16] Sebold F.J.G., Schuelter-Trevisol F., Nakashima L., Possamai Della Júnior A., Pereira M.R., Trevisol D.J. (2015). Electrocardiographic changes in adults living in a southern Brazilian city: A population-based study. Rev. Port. Cardiol..

[17] Silva M., Palhares D., Ribeiro L., Gomes P., Macfarlane P., Ribeiro A., Marcolino M. (2021). Prevalence of major and minor electrocardiographic abnormalities in one million primary care Latinos. J. Electrocardiol..

[18] Prineas R.J., Le A., Soliman E.Z., Zhang Z.-M., Howard V.J., Ostchega Y., Howard G. (2012). United States national prevalence of electrocardiographic abnormalities in black and white middle-age (45- to 64-Year) and older (≥65-Year) adults (from the Reasons for Geographic and Racial Differences in Stroke Study). Am. J. Cardiol..

[19] Abiodun A., Oladimeji A., Bamidele T., Adewole A., Mayowa O. (1970). Prevalence of ECG abnormalities among adults with metabolic syndrome in a Nigerian Teaching Hospital. Afr. Health Sci..

[20] Lohrmann G.M., Peters F., Srivathsan K., Essop M.R., Mookadam F. (2016). Electrocardiographic Abnormalities in Disease-Free Black South Africans and Correlations With Echocardiographic Indexes and Early Repolarization. Am. J. Cardiol..

[21] Pedro J.M., Rosário E., Brito M., Barros H. (2016). CardioBengo study protocol: a population based cardiovascular longitudinal study in Bengo Province, Angola. BMC Public Health.

[22] World Health Organization. The STEPS Instrument and Support Materials, 2015.

[23] R. Prineas, R.S. Crow, H.W. Blackburn, The Minnesota code manual of electrocardiographic findings: standards and procedures for measurement and classification. Boston, Mass. Editions : J. Wright, 1982.

[24] Gonçalves M.A.A., Pedro J.M., Silva C., Magalhães P., Brito M. (2020). Normal limits of the electrocardiogram in Angolans. J. Electrocardiol..

[25] Sellers M.B., Divers J., Lu L., Xu J., Smith S.C., Bowden D.W., Herrington D., Freedman B.I., Soliman E.Z. (2014). Prevalence and determinants of electrocardiographic abnormalities in African Americans with type 2 diabetes. J. Epidemiol. Glob. Health.

[26] Healy C.F., Lloyd-Jones D.M. (2016). Association of Traditional Cardiovascular Risk Factors with Development of Major and Minor Electrocardiographic Abnormalities: A Systematic Review. Cardiol. Rev..

[27] Dzudie A., Choukem S.-P., Adam A.K., Kengne A.P., Gouking P., Dehayem M. (2012). Prevalence and determinants of electrocardiographic abnormalities in sub-Saharan African individuals with type 2 diabetes: cardiovascular topic. Cardiovasc. J. Afr..

[28] Kumar A., Lloyd-Jones D.M. (2007). Clinical Significance of Minor Nonspecific ST-Segment and T-Wave Abnormalities in Asymptomatic Subjects: A Systematic Review. Cardiol. Rev..

[29] Greenland P., Xie X., Liu K., Colangelo L., Liao Y., Daviglus M.L., Agulnek A.N., Stamler J. (2003). Impact of minor electrocardiographic ST-segment and/or T-wave abnormalities on cardiovascular mortality during long-term follow-up. Am. J. Cardiol..

[30] Higgins I.T.T., Kannel W.B., Dawber T.R. (1965). The Electrocardiogram in Epidemiological Studies: Reproducibility, Validity, and International Comparison. J. Epidemiol. Community Heal.

[31] Ashley E.A., Raxwal V.K., Froelicher V.F. (2000). The prevalence and prognostic significance of electrocardiographic abnormalities. Curr. Probl. Cardiol..

[32] Kuroski de Bold M. (1999). Estrogen, natriuretic peptides and the renin–angiotensin system. Cardiovasc. Res..

[33] Bartel A. (1971). Electrocardiographic Predictors of Coronary Heart Disease. Arch. Intern. Med..

[34] van der Ende M.Y., Siland J.E., Snieder H., van der Harst P., Rienstra M. (2017). Population-based values and abnormalities of the electrocardiogram in the general Dutch population: The LifeLines Cohort Study. Clin. Cardiol..

[35] Camm A.J., Evans K.E., Ward D.E., Martin A. (1980). The rhythm of the heart in active elderly subjects. Am. Heart J..

[36] Demoulin R., Poyet R., Schmitt P., Sidibe S., Capilla E., Rohel G., Pons F., Jego C., Brocq F.X., Druelle A., Cellarier G.R. (2020). Particularités de l’électrocardiogramme du patient d’origine Africaine. Ann. Cardiol. Angeiol. (Paris).

[37] Walsh B., Macfarlane P.W., Prutkin J.M., Smith S.W. (2019). Distinctive ECG patterns in healthy black adults. J. Electrocardiol..

[38] Piwońska A., Piwoński J., Szcześniewska D., Drygas W. (2019). Population prevalence of electrocardiographic abnormalities: results of the Polish WAW-KARD study. Kardiol. Pol..

[39] Monin J., Bisconte S., Nicaise A., Hornez A.-P., Manen O., Perrier E. (2016). Prevalence of Intraventricular Conduction Disturbances in a Large French Population. Ann. Noninvasive Electrocardiol..

[40] Liao Y., Liu K., Dyer A., Schoenberger J.A., Shekelle R.B., Colette P., Stamler J. (1988). Major and minor electrocardiographic abnormalities and risk of death from coronary heart disease, cardiovascular diseases and all causes in men and women. J. Am. Coll. Cardiol..

[41] Yu L., Ye X., Yang Z., Yang W., Zhang B. (2020). Prevalences and associated factors of electrocardiographic abnormalities in Chinese adults: a cross-sectional study. BMC Cardiovasc. Disord..

[42] Bello-Sani F.F., Anumah F.E.O. (2009). Electrocardiographic abnormalities in persons with type 2 diabetes in Kaduna, Northern Nigeria. Int. J. Diabetes Metab..

[43] Olamoyegun A.M., Ogunmola O.O., Oladosu Y.T., Kolawole B.A. (2013). Int. Res. J. Med. Med. Sci..

[44] Machado D.B., Crow R.S., Boland L.L., Hannan P.J., Taylor H.A., Folsom A.R. (2006). Electrocardiographic findings and incident coronary heart disease among participants in the Atherosclerosis Risk in Communities (ARIC) study. Am. J. Cardiol..

[45] J.A. Walsh, R. Prineas, M.L. Daviglus, H. Ning, K. Liu, C.E. Lewis et al., Prevalence of electrocardiographic abnormalities in a middle-aged, biracial population: Coronary Artery Risk Development in Young Adults study, J. Electrocardiol. 43, 385.e1–9. https://doi.org/10.1016/j.jelectrocard.2010.02.001. 10.1016/j.jelectrocard.2010.02.001 PMC3569004 20374967

[46] Gepner A.D., Piper M.E., Leal M.A., Asthana A., Fiore M.C., Baker T.B. (2013). Electrocardiographic Changes Associated with Smoking and Smoking Cessation: Outcomes from a Randomized Controlled Trial. PLoS One.

[47] Oliveira A., Barros H., Júlia Maciel M., Lopes C. (2007). Tobacco smoking and acute myocardial infarction in young adults: a population-based case-control study. Prev. Med. (Baltim.).

[48] Daviglus M.L., Liao Y., Greenland P., Dyer A.R., Liu K., Xie X., Huang C.-F., Prineas R.J., Stamler J. (1999). Association of nonspecific minor ST-T abnormalities with cardiovascular mortality: the Chicago Western Electric Study. JAMA.

[49] Brink A.J. (1956). The normal electrocardiogram in the adult South African Bantu. S Afr. J. Lab Clin. Med..

[50] Sokolow M., Perloff D. (1961). The Prognosis of Essential Hypertension Treated Conservatively. Circulation.

