# Supplementary material for: Prevalence of major and minor electrocardiographic abnormalities and their relationship with cardiovascular risk factors in Angolans
Source: Int J Cardiol Heart Vasc. 2022 Feb 9;39:100965. doi: 10.1016/j.ijcha.2022.100965 (PMC8842030; doi:10.1016/j.ijcha.2022.100965)
Supplement: Supplementary data 1 [file mmc1.docx]

Table 1A – Characteristics of the participants with valid electrocardiogram at baseline (n = 2 379) (mean ± SD for continuous parameters, % for categorical parameters) stratified by gender

| Parameters | Male  n = 880 |  | Female  n = 1 499 | *p* | All  n = 2 379 |
| --- | --- | --- | --- | --- | --- |
| 15 – 29 years | 55.0 |  | 39.2 | <.001^¥^ | 45.0 |
| 30 – 44 years | 22.6 |  | 28.7 | .004^¥^ | 26.4 |
| 45 – 59 years | 16.4 |  | 26.1 | <.001^¥^ | 22.5 |
| ≥ 60 years | 6.0 |  | 6.1 | .998^¥^ | 6.1 |
| Age (years) | 32.0±14.4 |  | 37.01±14.4 | <.001* | 35.0±14.5 |
| Hypertension (%) | 17.0 |  | 21.0 | .017^‡^ | 19.1 |
| Diabetes (%) | 10.2 |  | 9.6 | .638^‡^ | 9.8 |
| Hypercholestrolemia (%) | 12.5 |  | 25.7 | <.001^‡^ | 21.1 |
| Obesity (%) | 14.9 |  | 32.1 | <.001^‡^ | **8.1** |
| Smoking (%) | 11.1 |  | 3.3 | <.001^‡^ | 6.2 |
| Sedentary (%) | 0.3 |  | 0.8 | <.001^‡^ | 0.6 |
| Alcohol consumption (%) | 43.8 |  | 31 | <.001¥ | 35.8 |
| Prior stroke or TIA (%) | 0.2 |  | 0.5 | .204^¥^ | 0.4 |
| Congestive heart failure | 0.5 |  | 0.2 | .258^¥^ | 0.3 |
| Ischemic heart disease (%) | 0.6 |  | 0.2 | .110¥ | 0.3 |

^¥^Test for the difference between two independent population proportions, *p*-values adjusted by the Bonferroni method;

*T-test for independent populations; ^‡^Test for the difference between two independent population proportions

Supplementary Table 1B. Prevalence of minor and major electrocardiographic abnormalities in detail stratified by gender

|  | **No. (%) of participants** | | | |
| --- | --- | --- | --- | --- |
| **Minnesota Code abnormalities** | **Male** | **Females** | ***p*** | **All** |
| **Minor abnormalities** | **272 (30.9)** | **259 (17.3)** |  | **531 (22.3)** |
| Sinus bradycardia | 22 (2.5) | 2 (0.13) | <.001 | 24 (1.01) |
| First degree AV block | 10 (1.14) | 7 (0.47) | .061 | 17 (0.72) |
| Ectopic atrial rhythm | 10 (1.14) | 13 (0.87) | .515 | 23 (0.97) |
| High T-wave amplitude | 7 (0.8) | 22 (1.47) | .149 | 29 (1.22) |
| Low QRS voltage in limbs leads | 3 (0.34) | 14 (0.93) | .096 | 17 (0.72) |
| Right atrial enlargement | 3 (0.34) | 4 (0.27) | .748 | 7 (0.29) |
| Poor R progression | 1 (0.11) | 0 (0.0) | .190 | 1 (0.04) |
| Minor isolated Q,QS waves | 2 (0.23) | 4 (0.27) | .849 | 6 (0.25) |
| Minor isolated ST abnormalities | 44 (5.0) | 57 (3.8) | .161 | 101 (4,25) |
| Wandering pacemaker | 1 (0.11) | 0 (0.0) | .190 | 1 (0.04) |
| Left axis deviation | 11 (1.25) | 13 (0.87) | .368 | 24 (1.01) |
| **Abnormal T wave inversion** |  |  |  |  |
| Anteroseptal site (V2–V4) | 14 (1.59) | 97 (6.47) | <.001 | 111 (4.67) |
| Lateral site (V5, V6, aVL e DI) | 1 (0.11) | 8 (0.53) | .107 | 9 (0.38) |
| Inferior site (DII, DIII e aVF) | 13 (1.48) | 34 (2.27) | .180 | 47 (1.98) |
| **Minor QT abnormalities** |  |  |  |  |
| QT interval low borderline | 3 (0.34) | 4 (0.27) | .748 | 7 (0.29) |
| Minor QT prolongation index (QTI≥112<116 or JTI if QRS≥120) | 1 (0.11) | 3 (0.2) | .617 | 4 (0.17) |
| **Incomplete intraventricular blocks** |  |  |  |  |
| Left anterior fascicular block | 3 (0.34) | 6 (0.40) | .818 | 9 (0.38) |
| Left posterior fascicular block | 0 (0.0) | 1 (0.07) | .441 | 1 (0.04) |
| Incomplete right bundle branch block | 5 (0.57) | 1 (0.07) | .018 | 6 (0.25) |
| **Premature beats** |  |  |  |  |
| Atrial or junctional premature beats | 18 (2.05) | 30 (2.0) | .944 | 48 (2.02) |
| Ventricular premature beats | 8 (0.91) | 22 (1.47) | .238 | 30 (1.26) |
| **Major abnormalities** | **N=28 (3.2)** | **N=81 (5.4)** |  | **109 (4.6)** |
| Left ventricular hypertrophy with major ST-T abnormalities | 12 (1.36) | 43 (2.87) | .018 | 55 (2.31) |
| Right Ventricular Hypertrophy | 1 (0.11) | 3 (0.2) | .617 | 4 (0.17) |
| Major Q-wave abnormalities (old MI) | 7 (0.8) | 10 (0.67) | .718 | 17 (0.72) |
| Major isolated ST-T abnormalities | 6 (0.68) | 2 (0.13) | .025 | 8 (0.34) |
| Major QT prolongation index (QT index ≥ 116%) | 2 (0.23) | 0 (0.0) | .064 | 2 (0.08) |
| **AV conduction defects** |  |  |  |  |
| Second or third degree atrioventricular block | 1 (0.11) | 0 (0.0) | .190 | 1 (0.04) |
| Wolff-Parkinson-White pattern | 0 (0.0) | 1 (0.07) | .441 | 1 (0.04) |
| **Ventricular conduction defect** |  |  |  |  |
| Complete right bundle branch block | 1 (0.11) | 5 (0.33) | .303 | 6 (0.25) |
| Complete left bundle branch block | 1 (0.11) | 6 (0.4) | .211 | 7 (0.29) |
| Nonspecific intraventricular conduction block | 1 (0.11) | 0 (0.0) | .190 | 1 (0.04) |
| Complete right bundle branch block, left anterior hemiblock | 2 (0.23) | 4 (0.27) | .849 | 6 (0.25) |
| Complete right bundle branch block, left posterior hemiblock | 0 (0.0) | 1 (0.07) | .441 | 1 (0.04) |
| **Arrhythmias** |  |  |  |  |
| Supraventricular Tachycardia | 1 (0.11) | 1 (0.07) | .703 | 2 (0.08) |
| Atrial fibrillation | 1 (0.11) | 1 (0.07) | .703 | 2 (0.08) |

*p*-values were obtained by the test for the difference between two independent population proportions
